# Effects of *Centella asiatica* (L.) Urb. on cognitive function and mood related outcomes: A Systematic Review and Meta-analysis

**DOI:** 10.1038/s41598-017-09823-9

**Published:** 2017-09-06

**Authors:** Panupong Puttarak, Piyameth Dilokthornsakul, Surasak Saokaew, Teerapon Dhippayom, Chuenjid Kongkaew, Rosarin Sruamsiri, Anchalee Chuthaputti, Nathorn Chaiyakunapruk

**Affiliations:** 1 Department of Pharmacognosy and Pharmaceutical Botany, Faculty of Pharmaceutical Sciences, Prince of Songkla University, Songkhla, Thailand; 2 Center of Pharmaceutical Outcomes Research, Department of Pharmacy Practice, Faculty of Pharmaceutical Sciences, Naresuan University, Phitsanulok, Thailand; 3 Department of Pharmacy Practice, Faculty of Pharmaceutical Sciences, Naresuan University, Phitsanulok, Thailand; 4 Center of Health Outcomes Research and Therapeutic Safety (Cohorts), School of Pharmaceutical Sciences, University of Phayao, Phayao, Thailand; 5 School of Pharmacy, Monash University Malaysia, Selangor, Malaysia; 6 Center of Excellence for Environmental Health and Toxicology, Naresuan University, Phitsanulok, Thailand; 7 Department for Development of Thai Traditional and Alternative Medicine, Ministry of Public Health, Nonthaburi, Thailand; 8 Asian Centre for Evidence Synthesis in Population, Implementation and Clinical Outcomes (PICO), Health and Well-being Cluster, Global Asia in the 21st Century (GA21) Platform, Monash University Malaysia, Bandar Sunway, Selangor, Malaysia; 9 School of Pharmacy, University of Wisconsin-Madison, Madison, WI USA

## Abstract

*Centella asiatica* (L.) Urb. has been used as an herbal brain tonic for mental disorders and enhancing memory, but no review of the overall evidence of *C. asiatica* and cognitive function has been conducted. This study aims to determine the effects of *C. asiatica* on cognitive function and its related properties. The current systematic review includes five randomized controlled trials (RCTs) conducted to determine the effect of *C. asiatica* alone and six RCTs conducted to determine the effect of *C. asiatica*-containing products. Meta-analysis indicated that there are no significant differences in all cognitive function domains of *C. asiatica* when compared to placebo. However, it could improve mood by increasing alert scores [SMD: 0.71 (95% CI; 0.01 to 1.41); I^2^ = 30.5%] and decreasing anger scores at 1 hour after treatment [SMD: −0.81 (95%CI; −1.51 to −0.09); I^2^ = 36.6%]. None of the studies reported adverse effects of *C. asiatica*. In conclusion, there is not strong evidence to support the use of *C. asiatica* for cognitive function improvement in each cognitive domain. *C. asiatica* could improve alertness and relieve anger. However, some limitations should be aware including dose regimen, plant preparation, standardization, and product variation. Future well-designed clinical trials using suitable doses of standardized *C. asiatica* are still needed.

## Introduction

Cognition can be defined as the group of mental processes that lead to knowledge through thought, experience, and the senses. Cognitive function consists of various domains including attention and concentration, executive function, information processing speed, language, visuospatial skill, working memory, verbal memory, and visual memory^1^. Diseases, drugs, chemicals, genetics, and aging can all cause declines in cognitive ability leading to cognitive impairment. Cognitive impairment may result in dementia or Alzheimer’s disease.

Acetylcholinesterase inhibitors (AChEIs) have been recommended as a first-line treatment for Alzheimer’s disease. However, AChEIs are also associated with various adverse events. To avoid these, herbal medicines such as Ginkgo (*Gingko biloba* L.)*, Curcuma longa* L.*, Melissa officinalis* L. and *Bacopa monnieri* L. Wettst have been increasingly used as alternatives to prevent or treat cognitive impairment^2–5^.

*Centella asiatica* (L.) Urban., (family Apiaceae), commonly known as asiatic pennywort or gotu kola, is a plant that has been used as an AChEI alternative. It is a perennial, herbaceous creeper with kidney shaped leaves commonly found and cultivated in Asian countries^6, 7^. It has been used since ancient times in Ayurvedic traditions under the name of mandukaparni^6–8^. This plant functions as an herb, spice, vegetable, and juice as well as in nutraceutical and cosmetic products. *C. asiatica* has been added to the Thailand National List of Essential Medicines for its antipyretic and wound healing properties^9^. It has also been selected as one of the five medicinal plants to be developed as a “champion herbal product” to generate income for the country^10^.

*C. asiatica* contains several active ingredients with the most important group being pentacyclic triterpenes, which includes asiaticoside, madecassoside, asiatic acid, and madecassic acid^8^. *C. asiatica* and its pentacyclic triterpenes are commonly used for their antipyretic, wound healing, anti-wrinkle, and anti-inflammation effects^11^. Important indications for *C. asiatica* in Ayurveda include its use for cognitive properties as a brain tonic, in the treatment of mental disorders, and as a memory-enhancing agent^6, 7, 12^. *C. asiatica* was shown to improve neuronal morphology and learning performance and enhance memory retention in animal models^13, 14^. Several mechanisms of action of *C. asiatica* were demonstrated for enhancing cognitive function, such as the inhibition of acetylcholinesterase activity, reduction of phospholipase A_2_ (PLA_2_) activity, protection against ß-amyloid formation, and protection from brain damage^15–17^. Furthermore, *C. asiatica* has also shown anti-stress, antidepressant, anxiolytic and anti-seizure properties in pre-clinical studies^18–20^. In animal models, asiaticoside and asiatic acid showed neuroprotective, antidepressive, and anxiolytic effects^20–23^. Learning and memory improvements facilitated by asiatic acid have been observed in passive and active avoidance tests^24^. From these data and its use in traditional medicine, *C. asiatica* is selected as one of the active ingredients in nutraceutical products for improving brain function.

A number of randomized controlled studies have investigated the clinical effect of *C. asiatica* on cognitive function^25, 26^. However, no study has summarized the overall evidence of *C. asiatica* on cognitive function and its related properties. Therefore, this study aims to systematically review all available evidence to determine the efficacy and safety of *C. asiatica* on cognitive function and its related properties including effects on mood and quality of life (QoL).

## Results

### Study selection

**Figure 1:**
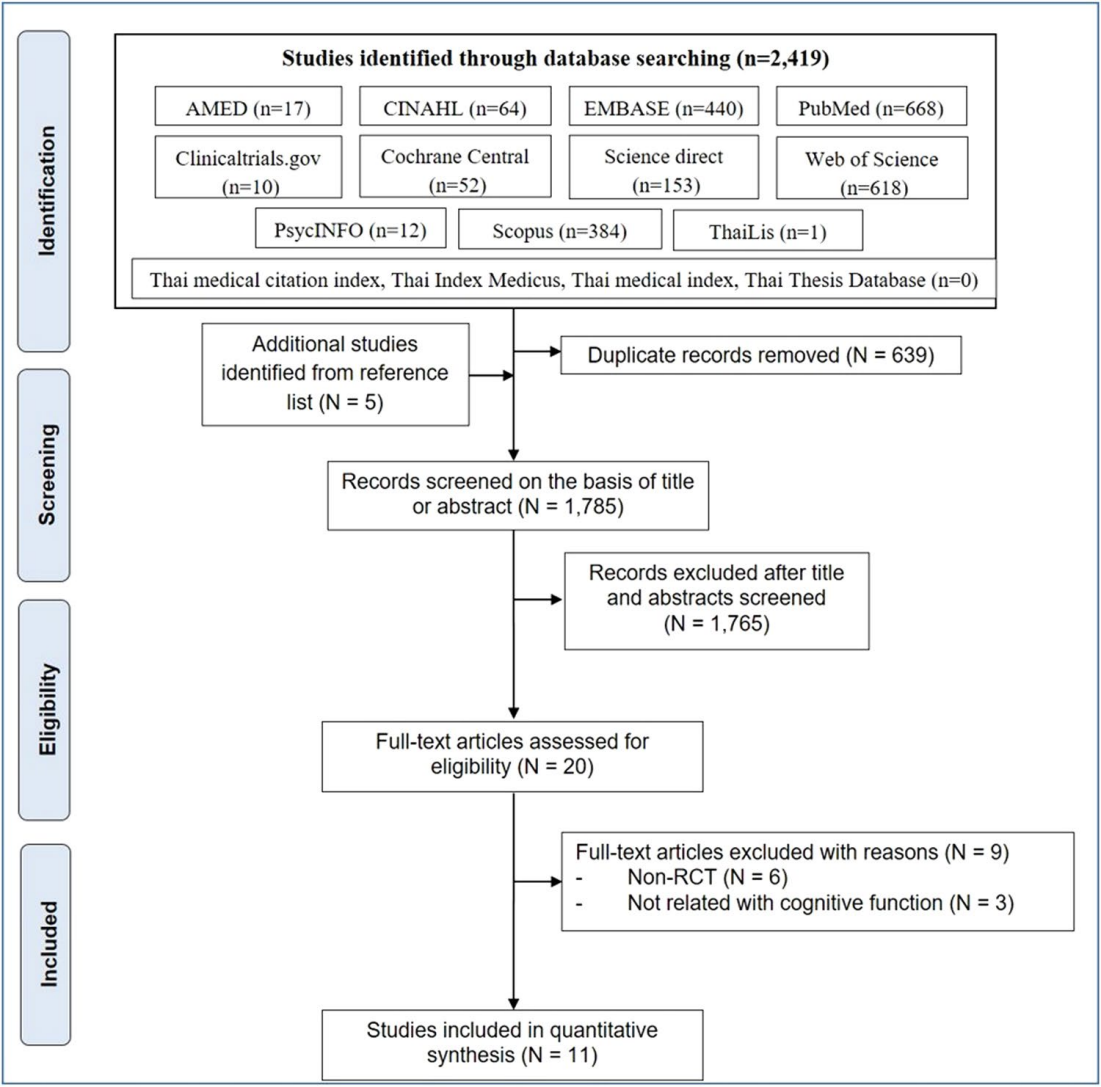
Flow of included studies.

A total of 2,419 articles were identified from the database searches, and five articles were added based on our review of the reference lists. Of the articles, 693 were excluded because of duplication. A total of 1,785 titles and abstracts were screened. Of the screened titles and abstracts, 20 full-text articles were reviewed, of which only 11 articles were included in the systematic review. The flow of included studies is depicted in Fig. 1.

### Study characteristics

Of the 11 included studies, five studies (45%) compared *C. asiatica* alone to placebo, and six studies (54%) compared a combination of *C. asiatica* versus other herbs. For combination products, three of the six studies (50%) used mix herbs as the active ingredients, two of the six studies (33%) used *Gingko biloba* as the major compound, and one study used a combination of vitamins and herbs (Table 1). Only one study (9%) did not report the Latin binomial nomenclature of the herbal ingredients^27^. Standardization methods were reported in three studies (27%)^26, 28, 29^ but only two studies quantitatively described the standardization^26, 29^. Nine studies (81%) were conducted using double-blind parallel designs, one used an open-labeled parallel design, and one used a cross-over design. Most studies (91%) were conducted in healthy volunteers, while one study was conducted in children with attention deficit hyperactive disorder. Other information (herbal supplement type, dosage form, plant preparation, dose of *C. asiatica*, standardization method, study characteristics, intervention and patient characteristics) is summarized in Table 1.

**Table 1:** Characteristics of *Centella asiatica* (L.) Urb. supplements and included studies.

Author	Herbal supplement type	Dosage form	Report Latin name (Raw material authentication)	Plant part	*C. asiatica* Preparation (solvent for extraction)	Standardization	Dose of *C. asiatica* per day (mg)	Standard compound content per day (mg)	Standard PT^#^ content per day (mg)	Pharmacy (P)/Manufacturer (M) production
Bradwejn, 2000^32^	Single	Mixture	Yes (No)	NR	Powder	NR	12,000	N/A	N/A	NR
Dev, 2009^25^	Single	Capsule	Yes (No)	NR	Powder	NR	5,000–8,000	N/A	N/A	Yes (M)
Mato, 2011^28^	Single	Capsule	Yes (Yes)	Aerial	Extract (water)	Standardized using TPC, AS, AA	250 500 750	TPC = 7.48, AS = 0.27, AA = 12.22 TPC = 14.19, AS = 0.55, AA = 24.45 TPC = 22.43, AS = 0.82, AA = 36.67	22.49 25.00 37.49	Yes (P)
Rao, 1977^35^	Single	Tablet	Yes (No)	NR	Powder	NR	500	N/A	N/A	NR
Wattanathorn, 2008^26^	Single	Capsule	Yes (Yes)	Aerial	Extract (water)	Standardized using TPC, AS, AA	250 500 750	TPC = 7.48, AS = 0.27, AA = 12.22 TPC = 14.19, AS = 0.55, AA = 24.45 TPC = 22.43, AS = 0.82, AA = 36.67	22.49 25.00 37.49	Yes (P)
Carlson, 2007^33^	Combination (*G. biloba* ^a^)	Softgel capsule	Yes (No)	NR	NR	NR	204	N/A	N/A	Yes (M)
Harris, 2011^27^	Combination (Vitamins^a^)	Tablet	No (No)	NR	Extract (NR)	NR	NR	N/A	N/A	Yes (M)
Katz, 2011^30^	Combination (Mix herb^a^)	Mixture	Yes (Yes)	NR	Extract (NR)	Standardized By Thin layer chromatography	NR	N/A	N/A	Yes (P)
Lewis, 2014^34^	Combination (*G. biloba* ^a^)	Capsule plus tablet	Yes (No)	Leaf	NR	NR	40	N/A	N/A	Yes (M)
Sarokte, 2013^29^	Combination (Mix herb^a^)	Powder	Yes (Yes)	NR	Powder	NR	1,000	N/A	N/A	NR
Udani, 2013^31^	Combination (Mix herb^a^)	Capsule	Yes (No)	NR	Extract (NR)	NR	100	N/A	N/A	Yes (M)
**Author**	**RCTs design**	**Participants**	**Inclusion age**	**Group**		**No. Participant**	**M:F**	**Mean age**	**Intake Duration**	**Interval Assessed**
Bradwejn, 2000^32^	DB, parallel	Healthy	18–45	*C. asiatica* 12 g single oral Placebo		20 20	21:19	NR	single oral	0, 30, 60, 90 min
Dev, 2009^25^	DB, parallel	Healthy	35–50	*C. asiatica* 3–4 g OD (50 mg/Kg) male *C. asiatica* 3–4 g OD (50 mg/Kg) female Placebo male Placebo female		10 11 9 10	10:0 0:11 9:0 0:10	43.3 ± 3.6 44.2 ± 5.9 40.1 ± 4.6 44.2 ± 4.8	60 days	0, 40, 60, 90 days
Mato, 2011^28^	DB, parallel	Healthy	55–80	*C. asiatica* extract 250 mg OD *C. asiatica* extract 500 mg OD *C. asiatica* extract 750 mg OD Placebo		20 20 20 20	1:19 1:19 1:19 1:19	64.6 ± 4.5 64.2 ± 5.1 66.8 ± 4.7 65.7 ± 4.8	90 days	0, 30, 60, 90, 120 days
Rao, 1977^35^	DB, parallel	Mentally retarded children	7–18	*C. asiatica* 500 mg OD Placebo		15 12	23:7	13.3	180 days	0, 90, 180 days
Wattanathorn, 2008^26^	DB, parallel	Healthy	Elderly	*C. asiatica* extract 250 mg OD *C. asiatica* extract 500 mg OD *C. asiatica* extract 750 mg OD Placebo		7 7 7 7	1:6 1:6 1:6 1:6	67.3 ± 1.4 62.0 ± 4.3 64.8 ± 2.7 65.9 ± 5.1	60 days	0, 60 min, 30, 60 days
Carlson, 2007^33^	DB, parallel	Healthy	65–85	*Ginkgo biloba* containing supplement (*C. asiatica* 68 mg/day)* Placebo		42 36	21:21 21:15	73.1 ± 4.8 72.1 ± 6.0	120 days	0, 120 days
Harris, 2011^27^	DB, parallel	Healthy man	50–69	Multivitamin + mineral + herb (*C. asiatica* 10–200 mg/day)* Placebo		25 25	25:0 25:0	62.1 ± 3.8 62.9 ± 7.0	56 day	0, 56 days
Katz, 2011^30^	DB, parallel	ADHD children	6–12	Compound herbal preparation (*C. asiatica* extract included)* Placebo		73 19	55:18 15:4	9.8 ± 1.6 9.4 ± 2.0	120 days	0, 120 days
Lewis, 2014^34^	DB, parallel	Healthy	60+	Ginkgo Synergy^®^ 2 cap* + Choline 4 tab (*C. asiatica* included) OPC Synergy^®^2 cap* + Catalyn^®^ 4 tab* Placebo		33 31 33	8:24 7:24 12:21	67.6 ± 6.3 68.5 ± 6.7 70.3 ± 8.3	90 day	0, 90, 180 days
Sarokte, 2013^29^	Open label, parallel	Healthy	10–16	MedhyaRasaya 4 g/day (*C. asiatica* 1 g/day) with milk Yogic practices Control (no intervention)		30 30 30	13:17 18:12 15:15	NR NR NR	90 day	0, 90 day
Udani, 2013^31^	DB, crossover	Healthy	35–65	SuperUlam* (*C. asiatica* extract 100 mg) single oral Placebo		20	10:10	47.7	single oral	0, 1, 2, 3, 4, 5 hours

NR = Not report, N/A = Not applicable, a = major component (as mentioned in article). UA = Ursolic acid, AS = Asiaticoside, AA = Asiatic acid, TPC = Total phenolic content. #PT = Pentacyclic triterpenes are consist of asiaticoside, asiatic acid and ursolic acid. DB = double blind, OD = once daily, ADHD = Attention deficit hyperactive disorder, *Commercial product, RCTs = Randomized controlled trials. NR = not report, M:F = Male:Female.

### Quality of included studies

Three of the studies (27%)^29–31^ had a high risk of bias, seven studies (64%) were unclear^25, 27, 28, 32–35^, and one study (9%) had a low risk of bias^26^. Although, all studies stated that they were randomized controlled trials, four of the trials (36%) were found to have unclear risk of bias for “sequence generation” because there was no description of the sequence generation methods. Most studies (72.7%) did not describe the “allocation concealment” method. In the bias domain of “blinding”, one study was an open-label study which was categorized as having a high risk of bias. All double-blind studies included had low risk. Furthermore, incomplete outcome data, selective outcome reporting, other sources of bias risk, and JADAD scores for each study are presented in Table 2.

**Table 2:** Methodological quality assessment of the included studies.

Author	Risk of bias domain								JADAD Score
	Sequence generation	Allocation concealment	Blinding		Incomplete outcome data	Selective outcome reporting	Other sources of bias	Overall risk of bias	
			Investigator	Participants					
*C. asiatica* alone									
Bradwejn, 2000^32^	U	U	L	L	L	L	L	U	3
Dev, 2009^25^	L	U	L	L	L	L	L	U	5
Mato, 2011^28^	L	U	L	L	L	L	L	U	5
Rao, 1977^35^	U	U	L	L	L	L	L	U	4
Wattanathorn, 2008^26^	L	L	L	L	L	L	L	L	5
Combination product contained with *C. asiatica*									
Carlson, 2007^33^	U	U	L	L	L	L	U	U	3
Harris, 2011^27^	U	U	L	L	L	L	L	U	4
Katz, 2010^30^	L	L	L	L	U	L	H	H	4
Lewis, 2014^34^	L	U	L	L	L	L	U	U	5
Sarokte, 2013^29^	L	U	H	H	L	L	L	H	1
Udani, 2013^31^	L	L	L	L	L	L	H	H	5

L = Low risk, U = Unclear, H = High risk.

### Effects of *C. asiatica* in cognitive function

Of the included studies, 60 cognitive function tests were described, but only 27 of the tests had sufficient data for a meta-analysis. The 27 cognitive function tests were each categorized into specific cognitive domains for the purpose of evaluating the cognitive improvement effect of *C. asiatica* ^1^. The domains included 1) overall cognitive status, 2) attention and concentration, 3) executive function, 4) working memory, 5) information processing speed, 6) language, 7) verbal memory, 8) visuospatial skill, and 9) visual memory (Table 3). The meta-analysis indicated no significant difference between *C. asiatica* and comparators (placebo) on any cognitive function domain [Overall cognitive status SMD: 0.49 (95%CI; −0.49 to 1.48), *I* ^2^ = 87.9%: Attention and concentration (score) SMD: 0.37 (95%CI; −0.48 to 1.22), *I* ^2^ = 77.0%: Attention and concentration (time) SMD: 0.01 (95%CI; −0.66 to 0.68), *I* ^2^ = 0.0%: Exclusive function (score) SMD: 0.17 (95%CI; −0.19 to 0.53*), I* ^2^ = 0.0%: Information processing (score) SMD: 0.51 (95%CI; −0.41 to 1.44), *I* ^2^ = 77.7%: Information processing (time) SMD: −0.23 (95%CI; −1.02 to 0.56), *I* ^2^ = 24.2%: Language SMD: 0.28 (95%CI; −0.62 to 1.17*), I* ^2^ = 83.0%: Visuospatial skill SMD: 0.61 (95%CI; −0.18 to 0.61), *I* ^2^ = 0.0%: Working memory (score) SMD: 0.61 (95%CI; −0.25 to 1.48*), I* ^2^ = 76.9%: Working memory (time) SMD: 0.61 (95%CI; −0.59 to 1.80*), I* ^2^ = 69.0%: Verbal memory SMD: 0.14 (95%CI; −0.43 to 0.71), *I* ^2^ = 61.6% and Visual memory SMD: 0.15 (95%CI; −0.28 to 0.58), *I* ^2^ = 22.1%]. All results are presented in Table 4. However, the findings in some trials indicated that *C. asiatica* alone may improve working memory. Significant positive effects were found on numeric working memory tests (Appendix D) (i.e., decreased working time) after patients received 750 mg (37.49 mg of pentacyclic triterpenes) of *C. asiatica* water extract for 1 hour [MD: 218.36 (95%CI; 39.73 to 397.0)]^26^. Moreover, the combination products also revealed possible effects on some cognitive function tests (Appendix D) associated with attention and concentration (overall attention test in attention deficit hyperactive disorder children) [MD: 16.8 (95%CI; 9.82 to 23.78)]^30^, executive function (trail making test B in healthy elderly participant) [MD: −16.92 (95%CI; −27.14 to −6.70)]^33^ and information processing speed (variability test in attention deficit hyperactive disorder children [MD: 23.90 (95%CI; 12.80 to 35.00)])^30^.

**Table 3:** Cognitive, mood, and quality of life tests included in the meta-analysis.

Function	Domain	Domain type	Test	Included studies
Cognitive	Overall cognitive status	Over all	Mini mental status examination (MMSE) Mini mental status examination (MMSE)	Carlson *et al*., 2007; Sarokte *et al*., 2013
			Intelligence quotient (IQ)	Rao *et al*., 1977
			Over all cognitive function	Udani, 2013
	Attention and concentration	Accuracy/Score	Digit vigilant test (accuracy)	Wattanathorn *et al*., 2008
			Sustained attention	Udani, 2013
			Broad attention	Dev *et al*., 2009
			Over all attention	Katz *et al*., 2010
		Time	Digit vigilant test (time)	Wattanathorn *et al*., 2008
			React time	Udani, 2013
	Executive function	Accuracy/Score	Symbol digit modalities	Carlson *et al*., 2007
			Executive process	Dev *et al*., 2009
		Time	Trail Making Test B (TMT-B)	Lewis *et al*., 2014
			Cognitive flexibility	Udani, 2013
	Information processing speed	Accuracy/Score	Processing speed	Dev *et al*., 2009
			Variability	Katz *et al*., 2010
		Time	Choice reaction time	Wattanathorn *et al*., 2008
			Processing speed	Udani, 2013
	Language	Over all	Controlled Oral Word Association test	Carlson *et al*., 2007; Lewis *et al*., 2014
	Visuospatial skill	Over all	Spatial memory (accuracy)	Wattanathorn *et al*., 2008
			Judgment of line orientation	Carlson *et al*., 2007
			Visual spatial thinking	Dev *et al*., 2009
	Working memory	Accuracy/Score	Numeric working memory (accuracy)	Wattanathorn *et al*., 2008
			Working memory	Dev *et al*., 2009
			Short term memory picture	Sarokte *et al*., 2013
		Time	Working memory	Udani, 2013
			Numeric working memory (time)	Wattanathorn *et al*., 2008
	Verbal memory	Over all	Word recognition (accuracy)	Wattanathorn *et al*., 2008
			Serial recall effect test - words	Sarokte *et al*., 2013
			List Learning	Carlson *et al*., 2007
	Visual memory	Over all	Picture recognition (accuracy)	Wattanathorn *et al*., 2008
			Benton Visual retention	Carlson *et al*., 2007
			Delayed recall	Dev, 2009
Mood	Mood	Over all	Profile of mood status (POMS) Profile of mood status (POMS) Mood rating	Udani, 2013; Harris *et al*., 2011; Bradwejn *et al*., 2000
	Mood scale	Over all	Bond-Lader mood scale Visual analog mood scale (VAMS)	Wattanathorn *et al*., 2008; Harris *et al*., 2011
Quality of life (QoL)	Total QoL	Over all	SF-36	Carlson *et al*., 2007
			General health questionnair	Harris *et al*., 2011
	Physical	Over all	SF-36 (physical function)	Mato *et al*., 2011
			Total physical	Udani, 2013

The same domain was pooled together for meta-analysis.

**Table 4:** Result of primary and secondary outcomes.

Domain	Inc. trial	N	Standardized mean difference [95% CI]	p-value	Heterogeneity (%*I* ^2^)	Pooled studies
**Primary outcomes**						
**Over all cognitive status**						
Outcomes at the end of study (All)	3	153	0.49 [−0.49, 1.48]	0.327	87.9	Rao *et al*., 1977; Carlson *et al*., 2007; Sarokte *et al*., 2013
Outcomes at the end of study (DB only)	2	93	−0.01 [−0.52, 0.51]	0.976	29.0	Rao *et al*., 1977; Carlson *et al*., 2007
Outcomes at the end of study (Combination only)	2	126	0.56 [−0.95, 2.08]	0.465	93.9	Carlson *et al*., 2007; Sarokte *et al*., 2013
Outcomes at the end of study (*C. asiatica* only)^#^	1	27	4.30 [−5.42, 14.02]	0.386	—	Rao *et al*., 1977
5 hr after ingestion (Combination only)^#^	1	20	−0.11 [−6.61, 4.51]	0.711	—	Udani, 2013
**Attention and concentration**						
**Attention (Score**)						
Outcomes at the end of study	3	146	0.37 [−0.48, 1.22]	0.395	77.0	Wattanathorn *et al*., 2008; Dev *et al*., 2009; Katz *et al*., 2010
1 month ingestion (*C. asiatica* only)	2	54	0.05 [−0.49, 0.58]	0.862	0.00	Wattanathorn *et al*., 2008; Dev *et al*., 2009
2 month ingestion (*C. asiatica* only)	2	54	−0.01 [−0.55, 0.52]	0.962	0.00	Wattanathorn *et al*., 2008; Dev *et al*., 2009
Outcomes at the end of study (Combinantion only)*^,#^	1	92	16.8 [9.82, 23.78]	0.000	—	Katz *et al*., 2010
1 hr after ingestion	2	34	−0.13 [−0.81, 0.54]	0.698	0.00	Wattanathorn *et al*., 2008; Udani, 2013
1 hr after ingestion (*C. asiatica* only)^#^	1	14	−4.76 [−34.90, 25.40]	0.757	—	Wattanathorn *et al*., 2008
1 hr after ingestion (Combination only)^#^	1	20	−1.25 [−11.12, 8.62]	0.804	—	Udani, 2013
**Attention (time)**						
1 hr after ingestion	2	34	0.01 [−0.66, 0.68]	0.977	0.00	Wattanathorn *et al*., 2008; Udani, 2013
1 hr after ingestion (*C. asiatica* only)^#^	1	14	6.88 [−38.74, 52.50]	0.758	—	Wattanathorn *et al*., 2008
1 hr after ingestion (Combination only)^#^	1	20	−0.90 [−9.34, 7.54]	0.834	—	Udani, 2013
**Executive function**						
**Executive function (Score)**						
Outcomes at the end of study	2	118	0.17 [−0.19, 0.53]	0.357	0.00	Carlson *et al*., 2007; Dev *et al*., 2009
Outcomes at the end of study (*C. asiatica* only)^#^	1	40	14.43 [−8.63, 37.49]	0.220	—	Dev *et al*., 2009
Outcomes at the end of study (Combination only)^#^	1	78	0.70 [−3.03, 4.43]	0.713	—	Carlson *et al*., 2007
**Executive function (Time)**						
5 hr after ingestion (Combination only)^#^	1	20	−3.25 [−10.53, 4.03]	0.381	—	Udani, 2013
Outcomes at the end of study (Combination only)*^,#^	1	48	−16.92 [−27.14, −6.70]	0.001	—	Lewis *et al*., 2014
**Information processing speed**						
**Information processing (Score)**						
Outcomes at the end of study	2	132	0.51 [−0.41, 1.44]	0.277	77.7	Dev *et al*., 2009; Katz *et al*., 2010
Outcomes at the end of study (*C. asiatica* only)^#^	1	40	0.49 [−7.63, 8.61]	0.906	—	Dev *et al*., 2009
Outcomes at the end of study (Combination only)*^,#^	1	92	23.90 [12.80, 35.00]	0.000	—	Katz *et al*., 2010
**Information processing (Time)**						
1 hr after ingestion	2	34	−0.23 [−1.02, 0.56]	0.572	24.2	Wattanathorn *et al*., 2008; Udani, 2013
1 hr after ingestion (*C. asiatica* only)^#^	1	14	36.97 [−134.2, 208.1]	0.672	—	Wattanathorn *et al*., 2008
1 hr after ingestion (Combination only)^#^	1	20	−6.25 [−15.63, 3.13]	0.192	—	Udani, 2013
**Language**						
Outcomes at the end of study (Combination only)^#^	2	126	0.28 [−0.62, 1.17]	0.545	83.0	Carlson *et al*., 2007; Lewis *et al*., 2014
**Visuospatial skill**						
Outcomes at the end of study	3	132	0.61 [−0.18, 0.61]	0.347	0.00	Carlson *et al*., 2007; Wattanathorn *et al*., 2008; Dev *et al*., 2009
Outcomes at the end of study (Healthy, elderly)	2	92	0.14 [−0.27, 0.55]	0.514	0.00	Carlson *et al*., 2007; Wattanathorn *et al*., 2008
Outcomes at the end of study (*C. asiatica* only)	2	54	0.30 [−0.24, 0.84]	0.279	0.00	Wattanathorn *et al*., 2008; Dev *et al*., 2009
**Working memory**						
**Working memory (Score)**						
Outcomes at the end of study	3	114	0.61 [−0.25, 1.48]	0.167	76.9	Wattanathorn *et al*., 2008; Dev *et al*., 2009; Sarokte *et al*., 2013
Outcomes at the end of study (*C. asiatica* only)	2	54	0.19 [−0.35, 0.72]	0.488	0.0	Wattanathorn *et al*., 2008; Dev *et al*., 2009
**Working memory (time)**						
1 hr after ingestion	2	34	0.61 [−0.59, 1.80]	0.319	69.0	Wattanathorn *et al*., 2008; Udani, 2013
1 hr after ingestion (*C. asiatica* only)*	1	14	218.36 [39.73, 397.0]	0.017	—	Wattanathorn *et al*., 2008
1 hr after ingestion (Combination only)	1	20	0.60 [−8.51, 9.71]	0.897	—	Udani, 2013
**Verbal memory**						
Outcomes at the end of study	3	152	0.14 [−0.43, 0.71]	0.635	61.6	Carlson *et al*., 2007; Wattanathorn *et al*., 2008; Sarokte *et al*., 2013
Outcomes at the end of study (Healthy, eldery)	2	92	−0.15 [−0.56, 0.26]	0.473	0.00	Carlson *et al*., 2007; Wattanathorn 2008
Outcomes at the end of study (Combination only)	2	138	0.23 [−0.51, 0.97]	0.543	78.8	Carlson *et al*., 2007; Sarokte *et al*., 2013
Outcomes at the end of study (*C. asiatica* only)	1	14	−2.07 [12.26, 8.12]	0.691	—	Wattanathorn *et al*., 2008
**Visual memory**						
Outcomes at the end of study	3	132	0.15 [−0.28, 0.58]	0.487	22.1	Carlson *et al*., 2007; Dev *et al*., 2009; Wattanathorn *et al*., 2008
Outcomes at the end of study (*C. asiatica* only)	2	54	0.37 [−0.24, 0.98]	0.235	18.8	Wattanathorn *et al*., 2008; Dev *et al*., 2009
**Secondary outcomes**						
**Mood (self-report from participants)**						
**Bond-Lader mood scale/VAMS**						
Outcomes at the end of study (Alert)*	2	64	0.71 [0.01, 1.41]	0.046	30.5	Wattanathorn *et al*., 2008; Harris *et al*., 2011
Outcomes at the end of study (Alert) (*C. asiatica* only)*^,#^	1	14	9.38 [1.71, 17.05]	0.017	—	Wattanathorn *et al*., 2008
Outcomes at the end of study (Alert) (Combination only)^#^	1	50	7.20 [−0.98, 15.38]	0.085	—	Harris *et al*., 2011
Outcomes at the end of study (Content)	2	64	0.30 [−0.19, 0.80]	0.227	0.00	Wattanathorn *et al*. 2008; Harris *et al*., 2011
Outcomes at the end of study (Content) (*C. asiatica* only)^#^	1	14	2.38 [−2.77, 7.53]	0.365	—	Wattanathorn *et al*., 2008
Outcomes at the end of study (Content) (Combination only)^#^	1	50	3.90 [−4.57, 12.37]	0.367	—	Harris *et al*., 2011
Outcomes at the end of study (Clam)	2	64	0.60 [−0.30, 1.50]	0.194	53.5	Wattanathorn *et al*., 2008; Harris *et al*., 2011
Outcomes at the end of study (Clam)* (*C. asiatica* only)^#^	1	14	2.37 [0.33, 4.41]	0.023	—	Wattanathorn 2008
Outcomes at the end of study (Clam) (Combination only)^#^	1	50	3.60 [−4.19, 11.39]	0.365	—	Harris *et al*., 2011
**POMS and mood rating (self-report from participants)**						
**Tension**						
1 hr after ingestion	2	59	−0.05 [−0.56, 0.46]	0.846	0.00	Bradwejn *et al*., 2000; Udani, 2013
2 hr after ingestion	2	59	0.30 [−0.99, 1.58]	0.651	80.8	Bradwejn *et al*., 2000; Udani, 2013
Outcomes at the end of study (Combination only)^#^	1	50	−1.70 [−4.62, 1.22]	0.253	—	Harris *et al*., 2011
**Depression**						
1 hr after ingestion	2	59	0.09 [−1.53, 1.71]	0.916	87.8	Bradwejn *et al*., 2000; Udani, 2013
2 hr after ingestion	2	59	0.33 [−1.42, 2.08]	0.710	89.0	Bradwejn *et al*., 2000; Udani, 2013
Outcomes at the end of study (Combination only)^#^	1	50	−1.00 [−5.05, 3.05]	0.628	—	Harris *et al*., 2011
**Angor**						
1 hr after ingestion*	2	59	−0.81 [−1.51, −0.09]	0.026	36.6	Bradwejn *et al*., 2000; Udani, 2013
2 hr after ingestion	2	59	0.27 [−0.35, 0.89]	0.386	26.4	Bradwejn *et al*., 2000; Udani, 2013
Outcomes at the end of study (Combination only)^#^	1	50	−2.90 [−7.29, 1.49]	0.196	—	Harris *et al*., 2011
**Vigor**						
1 hr after ingestion	2	59	−0.25 [−1.68, 1.19]	0.737	85.0	Bradwejn *et al*., 2000; Udani, 2013
2 hr after ingestion	2	59	−0.16 [−1.10, 0.78]	0.735	66.5	Bradwejn *et al*., 2000; Udani, 2013
Outcomes at the end of study (Combination only)^#^	1	50	0.70 [−2.88, 4.28]	0.701	—	Harris *et al*., 2011
**Fatigue**						
1 hr after ingestion	2	59	0.39 [−0.42, 1.20]	0.345	54.1	Bradwejn *et al*., 2000; Udani, 2013
2 hr after ingestion	2	59	0.26 [−0.53, 1.05]	0.640	52.6	Bradwejn *et al*., 2000; Udani, 2013
Outcomes at the end of study (Combination only)^#^	1	50	−1.30 [−4.06, 1.46]	0.355	—	Harris *et al*., 2011
**Confusion**						
1 hr after ingestion	2	59	−0.48 [−1.65, 0.70]	0.427	76.6	Bradwejn *et al*., 2000; Udani, 2013
2 hr after ingestion	2	59	0.11 [−0.40, 0.62]	0.675	0.00	Bradwejn *et al*., 2000; Udani, 2013
Outcomes at the end of study (Combination only)^#^	1	50	−0.90 [−3.26, 1.46]	0.454	—	Harris *et al*., 2011
**Quality of life**						
Outcomes at the end of study (Physical)	2	60	0.21 [−0.30, 0.72]	0.417	0.00	Mato *et al*., 2011; Harris *et al*., 2011
Outcomes at the end of study (Total)	2	128	0.04 [−0.87, 0.95]	0.931	84.4	Carlson *et al*., 2007; Harris *et al*., 2011

^*^Significant (p < 0.05).

#Presented as mean difference (not standardized mean difference).

Combination only = Only combination product, *C. asiatica* only = *C. asiatica* alone product.

Outcomes at the end of study = Outcomes measured at the longest following up.

*Significant (p < 0.05), CI = confident interval.

#Presented as mean difference (not standardized mean difference).

All = pooled all data, Combination only = Only combination product, *C. asiatica* only = *C. asiatica* alone product, DB = Double blind, Score = Score unit, Time = Time unit, Healthy = Healthy volunteer, Elderly = elderly volunteer.

Outcomes at the end of study = Outcomes measured at the longest following up time.

For secondary outcomes, *C. asiatica* could increase self-reported alert scores [SMD: 0.71 (95%CI; 0.01 to 1.41), *I* ^2^ = 30.5%]. Furthermore, ingestion of *C*. a*siatica* water extract (750 mg/day) for 2 months showed an increase in self-reported alertness [MD: 9.38 (95%CI; 1.71 to 17.05)] and self-reported calmness [MD: 2.37 (95%CI; 0.33 to 4.41)]*. C. asiatica* also decreased self-reported anger scores at 1 hour after treatment [SMD: −0.81 (95%CI; −1.51 to −0.09), *I* ^2^ = 36.6*%*]. However, no other significant differences for mood or quality of life could be identified. Other findings of all outcomes are presented in Table 4.

### Adverse effects

Adverse effects or toxicity associated with *C. asiatica* were also evaluated based on the included articles. No adverse effects were reported in any studies looking at *C. asiatica* alone. However, for studies of combination products, four studies reported mild adverse events of *C. asiatica*-containing products. Two studies reported adverse event rates comparable to the placebo rate^31, 34^, while another two studies reported lower rates of adverse event for *C. asiatica*-containing products^30, 33^. Common adverse events were gastrointestinal discomfort, flatulence, nausea, headache, decreased appetite, sedation, and rash. Hepatotoxicity, which has been reported in one previous case report^36^, was not observed in any of the included RCTs.

## Discussion

This systematic review and meta-analysis provides a comprehensive summary of the effects of *C. asiatica* on cognitive function.

Current evidence does not support the effects of *C. asiatica* alone on overall cognitive function. However, ingestion of *C. asiatica* water extract (750 mg/day) for 1 hour may improve working memory, as shown in the positive effect on the numeric working memory test^26^ by a decrease in working time. This finding does not agree with a recent quasi-experimental study which found a statistically significant improvement in the memory domain of patients who had vascular cognitive impairment treated with *C. asiatica* extract (1,000 mg/day) when compared to patients treated with 3 mg/day of folic acid^37^. In that study, however, the dose of *C. asiatica* was higher than nine of the eleven trials included in this meta-analysis. Thus, the non-significant differences in overall cognitive function between *C. asiatica* and its comparators observed in this review might be due to the dosages used in the included studies. In traditional use and experimental evidence^38^, at least 3 grams of *C. asiatica* needs to be used to improve cognitive function. However, only two included studies^29, 32^ used doses greater than 3 g of *C. asiatica* per day, while the rest used lower doses.

The combination of *C. asiatica* with other herbs also showed non-significant improvements in overall cognitive function. However, the combination products in other studies have revealed that there arepossible effects on attention and concentration^30^, executive function^34^ and information processing speed^30^. The improvement in cognitive function from the combination products might be due to the synergistic effects of *C. asiatica* with other herbs or the effects of other herbs in *C. asiatica*-containing products such as *G. biloba* ^31, 34^. *G. biloba* is a well-known herbal medicine used for cognitive impairment. From previous systematic reviews and meta-analyses, *G. biloba* exhibited potential benefits for cognitive improvement in mild cognitive impairment or Alzheimer’s patients^2, 5^. Moreover, beneficial effects on cognitive function of *Withania somnifera* (L.) Dunal, Spirulina (*Arthrospira platensis)* and paeoniflorin (monoterpene glucoside) have been exhibited in different pre-clinical models^30^. None of the studies reported details on which parts of *C. asiatica* were used in the combination or how the combinations were prepared. Thus, thefindings could not show the direct effect of *C. asiatica* on cognitive function, and there is currently a lack of persuasive evidence to confirm a cognitive enhancing effect of *C. asiatica*.

For secondary outcomes, *C. asiatica* consumption was associated with improvements in self-reported alertness (after 2 months of ingestion) and with reductions in self-reported anger (after 1 hour of ingestion). Moreover, *C. asiatica* alone (750 mg/day for 2 months) induced alertness and calmness. These improvements in alertness and calmness may facilitate cognitive function by improving working memory, attention and concentration, executive function and information processing speed, and memory capacity and by reducing the time to solve problems. These results also support the traditional use of *C. asiatica* as a brain tonic. However, the positive effects may be caused by the other herbs in the combination products, so firm conclusions on the efficacy of *C. asiatica* cannot be drawn. There were also no significant differences between *C. asiatica* and placebo for physical or total QoL scores. From the safety data, *C. asiatica* seems to be safe since there were no serious adverse events reported in any of the included articles.

This meta-analysis included both *C. asiatica* alone and *C. asiatica* combined with other herbs. There were differences among the included studies such as differences in the part of *C. asiatica* used, dosage forms, extraction procedures, preparation, and outcome measurements. However, based on the objectives, all cognitive function data were collected from the RCTs that used any type of *C. asiatica*. The authors believe that the analysis is valid to address the objectives. Using the standardized mean difference (SMD), allowed the effect of *C. asiatica* on cognitive function to be assessed across the various types of cognitive function measurements used in the included studies. SMD converts data from different scales to a common scale. However, the standardization causes the original information for each measurement to be lost, so the findings cannot be interpreted in common units. They can only provide the level of significance of the effect of *C. asiatica* compared to the comparators^39^.

A classification defined by previous studies was used to determine the domains of cognitive function and pool the findings together^1, 37^. This classification has been used in several studies^37, 40–42^ to classify the domains of cognitive function and pool their findings. Thus, it is believed that the approach is appropriate for this meta-analysis. As no validation study of the classification was conducted, future studies may look into this issue.

This review identified limitations in the reporting of clinical studies of *C. asiatica*. Most of the included studies did not report details on the parts of the *C. asiatica* plant used in the products, the standardization methods, the active marker contents, or the methods for preparing the products. Only three of eleven (27*%)* trials^26, 28, 30^ reported standardization methods of the plant extract, and only two trials (18*%)* reported the amount of the active compounds (asiaticoside and asiatic acid) contained in the *C. asiatica* extract^26, 28^. Moreover, none of the studies reported qualitative analyses (such as HPLC fingerprints) of the *C. asiatica* in their studies. Clinical trials of herbal medicine should use standardized products as interventions and should report the detail of each intervention according to the CONSORT statement for reporting herbal medicinal interventions^43^. Furthermore, the place, conditions, and season of cultivation as well as the parts of the plant used can affect the pentacyclic triterpene (asiaticoside, asiatic acid, madecassoside, madecassic acid) contents of the *C. asiatica* raw material^44^. Lack of herbal standardization in clinical trials may affect the quality of studies and explain the variations in the clinical effects across studies. Interpretation of the findings of this systematic review should be done with cautions due to the lack of information about standardization.

Another consideration is that the doses of *C. asiatica* in each study were different, ranging from 40–12,000 mg/day. Variations in *C. asiatica* preparation were also observed. For *C. asiatica* alone, three of five trials used dry *C. asiatica* powder ranging from 500–12,000 mg/day while two trials used *C. asiatica* water extract ranging from 250–750 mg/day. Furthermore, the doses used in most of the included studies were lower than the traditional dose recommendation for cognitive improvement (3 g/day of *C. asiatica* powder)^38^. In the combination products, the dose of *C. asiatica* was very low (40–204 mg/day) compared with the main active component except in one study that used *C. asiatica* 1,000 mg/day^29^. Additionally, the dose and preparation of *C. asiatica* in some combination products was not clear. These limitations may affect the pooled data of *C. asiatica* in each cognitive domain. Moreover, the observed findings did not support a direct effect of *C. asiatica* containing products on cognitive function. There is currently a lack of persuasive evidence to confirm a cognitive enhancing effect of *C. asiatica*.

Based on this review, future well-designed clinical trials are warranted to evaluate the effects of *C. asiatica* products on cognitive function and mood as well as its safety. Standardized doses of *C. asiatica* products should be investigated over short-term and long-term periods of ingestion for effects in each specific cognitive domain, especially working memory, attention and concentration, executive function, and information processing speed.

In conclusion, the findings revealed that there is no strong evidence to support the effect of *C. asiatica* on overall cognitive function improvement. However, *C. asiatica* may improve working memory. A combination of *C. asiatica* with other herbs may improve attention and concentration, executive function, and information processing speed. *C. asiatica* may also improve mood disorders in terms of self-reported alertness and reductions in self-reported anger. Issues with dosage and preparation standardization need to be considered when these findings are applied. Future well-designed clinical trials are needed to assess the effects of standardized *C. asiatica* on cognitive function and mood as well as safety.

## Methods

This systematic review was conducted according to the Cochrane Collaboration framework guidelines^39^ and was reported in accordance with the Preferred Reporting Items for Systematic Reviews and Meta-Analyses (PRISMA) Statement^45^. The review protocol was registered with PROSPERO (registration number: CRD42015023595).

### Search strategies and study selection

An electronic search was conducted for original articles from inception to September 2016 using a number of electronic databases including AMED, CINAHL, Cochrane Central Register of clinical trial, EMBASE, PubMed, Psyc﻿info, Science direct, Scopus, www.clinicaltrials.gov, ThaiLis, Thai Index Medicus, Thai Medical Index, and Thai Thesis Database. Strategic search terms were *C. asiatica* name, OR active compound from *C. asiatica* (such as asiaticoside, madecassoside, asiatic acid, madecassic acid), OR *C. asiatica* containing products combined with cognitive function or memory and its related properties including mood and quality of life. Details of the search strategies are described in appendix A. Eligibility criteria were 1) published and unpublished randomized controlled trials in patients or healthy volunteers and 2) reported effects of *C. asiatica* or a combination of *C. asiatica* with other herbs in humans. No language restriction was applied. To ensure that the search would be thorough, reference lists were reviewed to identify potential studies not indexed in above mentioned databases. Furthermore, corresponding authors of identified studies were consulted for additional studies as sources. Titles and abstracts were screened according to the eligibility criteria. Full-text articles of the potential studies were retrieved from database or corresponding authors and were subsequently assessed independently by two researchers (PP, PD) for inclusion in the meta-analysis. Disagreements between the independent researchers were settled by discussion and consensus with a third independent researcher (NC).

### Data extraction and quality assessment

Data extraction was undertaken using a standard data extraction form. Extracted data included study design, characteristics of participants, characteristics of intervention and comparator, duration of herbal use, follow-up time, cognitive function tests, and cognitive function outcomes. Data for cognitive function tests included the name of the cognitive function test, the cognitive function domain, the outcome measures, and the outcome scale. For this meta-analysis, each cognitive test was categorized into one specific domain of cognitive function following a previous study^1^. This approach avoids over-weighting effects and provides consistency for the evaluation of the effect of *C. asiatica* on cognition across studies (Appendix B, C). A primary outcome of interest was the clinical effect of *C. asiatica* on cognitive function in each domain (Appendix B) including attention and concentration, executive function, information processing speed, language, visuospatial skill, working memory, verbal memory, and visual memory as well as overall cognitive status. In addition, secondary outcomes were mood, quality of life, and adverse events reported across each intervention. Where relevant data were unavailable, it was sought directly from the corresponding authors.

The quality of included studies was assessed using the Cochrane risk of bias tool^39^ and JADAD score^46^. Sequence generation, allocation concealment, blinding, incomplete outcome data, selective outcome reporting, and other sources of bias were evaluated. Data search, data extraction, and quality assessment were performed by PP and PD. Disagreements between the reviewers were settled through discussion and consensus.

### Statistical analysis

To determine the cognitive effect of *C. asiatica*, data for individual cognitive function tests were compared between *C. asiatica* and its comparator using standardized mean difference (SMD) or mean difference (MD) with a 95% confidence interval (CI). Heterogeneity was assessed by the *I* ^2^-statistic^47^. Thresholds of *I* ^2^ were interpreted in accordance with the magnitude and direction of effects and strength of evidence of heterogeneity. *I* ^2^ values of more than 50% indicated substantial heterogeneity. Data from included studies were pooled using the Der Simonian and Laird random-effects model^48^. The software used for data analysis was STATA version 12 (STATA Corp, College Station, TX, USA).

### Data availability

The datasets generated during and/or analyzed during the current study are available from the corresponding author on reasonable request.
